# Impact of Point-of-Care Ultrasound-Guided Resuscitation Protocols in the Treatment of Septic Shock: A Systematic Review of Hemodynamic Outcomes and Mortality

**DOI:** 10.7759/cureus.100850

**Published:** 2026-01-05

**Authors:** Alexis Agustin A Dunay Silva, Santiago Medina Tovar, Juan Sebastian Usma Gutierrez, Rashell Danae Fiallos Baldeón, Elvis Roque Arias Merino, Montserrat Ceja, Pablo Andrés Gutiérrez Hoyos

**Affiliations:** 1 Internal Medicine Department, Hospital Barros Luco Trudeau, San Miguel, CHL; 2 Emergency Department, Universidad Cooperativa de Colombia, Villavicencio, COL; 3 Medicine Department, Universidad Antonio Nariño, Bogotá, COL; 4 Medicine Department, Universidad Regional Autónoma de los Andes, Ambato, ECU; 5 Medicine Department, Universidad Internacional del Ecuador, Quito, ECU; 6 Medicine Department, Universidad Autónoma de Guadalajara, Guadalajara, MEX; 7 Medicine Department, Institución Prestadora de Servicios (IPS) Salud en Casa SURA, Cali, COL

**Keywords:** fluid resuscitation, hemodynamic monitoring, mortality, point-of-care ultrasound (pocus), septic shock

## Abstract

Septic shock is a life-threatening condition requiring rapid hemodynamic stabilization. This review aimed to evaluate the impact of point-of-care ultrasound (POCUS)-guided resuscitation on mortality and hemodynamic outcomes in adults with septic shock.

Following Preferred Reporting Items for Systematic Reviews and Meta-Analyses (PRISMA) 2020 guidelines, PubMed, the Cochrane Library, and Google Scholar were searched up to October 2025 for studies comparing POCUS-guided to conventional resuscitation. Study quality was assessed using the Cochrane Risk of Bias 2.0 and Newcastle-Ottawa scale (NOS) tools. Due to heterogeneity in study designs and outcome measures, a narrative data synthesis was performed, summarizing findings across mortality, hemodynamic, fluid balance, and organ function domains.

Fourteen studies met the inclusion criteria, encompassing 11 randomized controlled trials (RCTs) and three cohort studies. Ultrasound-guided resuscitation protocols demonstrated improvements in hemodynamic stability, including higher mean arterial pressure, improved oxygenation indices, and greater lactate clearance compared to standard care. Fluid management outcomes indicated lower total fluid administration and more favorable fluid balance. Several studies reported reductions in organ dysfunction markers, the earlier restoration of perfusion, and the decreased incidence of acute kidney injury (AKI). Additionally, ultrasound-guided protocols were associated with shorter durations of mechanical ventilation and reduced intensive care unit (ICU) and hospital stays. Mortality outcomes showed variable results, with some evidence suggesting a potential survival benefit.

Ultrasound-guided resuscitation improves hemodynamic optimization, fluid balance, and organ recovery, offering a more individualized approach to septic shock management.

## Introduction and background

Septic shock is a severe subtype of sepsis, which is a life-threatening organ dysfunction caused by a dysregulated host response to infection [1]. Shock is broadly classified into hypovolemic, cardiogenic, obstructive, and distributive phenotypes, each characterized by distinct hemodynamic mechanisms. Septic shock represents the most common form of distributive shock and is uniquely characterized by peripheral vasodilation, relative or absolute hypovolemia, myocardial depression, and profound microcirculatory dysfunction, often coexisting simultaneously and evolving dynamically during resuscitation. With an estimated 30 million episodes and six million fatalities each year, septic shock has garnered significant interest due to its high morbidity and mortality [2]. Despite the development of new treatments, successful therapy remains challenging [3]. On the other hand, comprehensive studies on severe sepsis have led to a better understanding of the pathophysiology and management of septic shock. To enhance outcomes, recommendations for the treatment of septic shock were published in 2003 through collaborative research in infectious diseases and critical care [4].

One of the most widely used strategies in the management of septic shock is fluid resuscitation. Early goal-directed therapy (EGDT) was recommended during the first six hours following septic shock in the 2004 Surviving Sepsis Campaign (SSC) guidelines [4]. EGDT aims to improve tissue perfusion by targeting predefined hemodynamic parameters through fluid resuscitation, vasopressors, and inotropic support [5]. However, septic shock differs from other shock phenotypes in that excessive fluid loading may exacerbate endothelial injury, capillary leak, pulmonary edema, and right ventricular dysfunction, making a uniform resuscitation approach potentially harmful. Evidence has emerged suggesting that standard care approaches do not consistently translate into improved outcomes [6,7]. For instance, restrictive fluid strategies have been associated with reduced need for vasoactive medications and mechanical ventilation, and several studies demonstrate that early large-volume crystalloid administration does not improve prognosis and may increase complications [8-10]. Nevertheless, the 2021 SSC guidelines continue to recommend the intravenous administration of at least 30 mL/kg of crystalloids within the first three hours of resuscitation, albeit with the acknowledgment of low-quality evidence [11].

To better individualize resuscitation, the use of noninvasive hemodynamic assessment tools, such as echocardiography, is increasingly advocated [12]. Point-of-care ultrasound (POCUS), including focused cardiac ultrasound (FoCUS), has emerged as a bedside modality capable of real-time, dynamic assessment of cardiac function, preload responsiveness, venous congestion, and pulmonary edema through the evaluation of ventricular function, inferior vena cava (IVC) respiratory variability, and lung B-lines. However, the comparative effectiveness of standard treatment strategies versus ultrasound-guided fluid resuscitation remains debated [13]. Kaselitz and Seymour emphasize that POCUS is particularly valuable in patients with undifferentiated hypotension or septic shock by helping clinicians identify distributive shock patterns, detect septic cardiomyopathy, and distinguish patients likely to benefit from further fluid administration from those at high risk of fluid overload [14]. In studies evaluating its diagnostic utility in distributive shock, POCUS combined with clinical assessment demonstrated sensitivities ranging from 63.6% to 75% and specificities of 99.7% to 100%, highlighting its role as an adjunctive decision-making tool rather than a replacement for clinical judgment. In a single-center randomized trial of emergency department (ED) patients with septic shock, the POCUS-guided assessment of IVC respiratory variation resulted in a reduction of approximately 700 mL of intravenous fluid administered within the first six hours compared to usual care, without a significant difference in 30-day mortality.

A recent systematic review and meta-analysis by Basmaji et al. evaluated the impact of POCUS-guided resuscitation on clinical outcomes in adult patients with shock, including 18 randomized controlled trials (RCTs) [15]. POCUS-guided resuscitation was associated with a reduction in 28-day mortality (relative risk {RR}, 0.88; 95% CI, 0.78-0.99), a shorter duration of vasoactive medication use (mean difference, -0.73 days; 95% CI, -1.16 to -0.30), and a lower requirement for renal replacement therapy (relative risk, 0.80; 95% CI, 0.63-1.02), with low to moderate certainty of evidence. Improvements in lactate clearance were also observed with high-certainty evidence, while effects on intensive care unit (ICU) length of stay, mechanical ventilation, and hospital admission rates were minimal or uncertain. However, a key limitation of this meta-analysis is that it pooled all shock etiologies, including cardiogenic, hypovolemic, and obstructive shock, without isolating septic shock as a distinct subgroup. Given the unique and heterogeneous hemodynamic profile of septic shock, the extrapolation of these findings to this population may be limited. Therefore, the present systematic review aims to focus exclusively on the impact of POCUS-guided resuscitation protocols in patients with septic shock, providing a targeted synthesis of evidence related to mortality, hemodynamic optimization, organ support requirements, and fluid management outcomes in this critically ill cohort.

## Review

Methodology

Study Design

This systematic review was conducted following the Preferred Reporting Items for Systematic Reviews and Meta-Analyses (PRISMA) 2020 guidelines [16]. The review aimed to evaluate the impact of point-of-care ultrasound (POCUS)-guided resuscitation protocols on hemodynamic outcomes and mortality in adult patients with septic shock.

Search Strategy

A comprehensive literature search was conducted in PubMed, the Cochrane Library, ScienceDirect, and Google Scholar from database inception to October 2025. The search strategy combined controlled vocabulary and free-text terms related to sepsis, septic shock, fluid resuscitation, hemodynamic monitoring, and point-of-care ultrasound.

For PubMed, the following search string was used: (("point-of-care ultrasound"[Title/Abstract] OR "POCUS"[Title/Abstract] OR "ultrasound-guided"[Title/Abstract] OR "echocardiography"[Title/Abstract] OR "lung ultrasound"[Title/Abstract] OR "integrated cardiopulmonary ultrasound"[Title/Abstract] OR "VExUS"[Title/Abstract] OR "IVC ultrasound"[Title/Abstract]) AND ("septic shock"[Title/Abstract] OR "sepsis"[Title/Abstract] OR "sepsis shock"[Title/Abstract] OR "severe sepsis"[Title/Abstract]) AND ("resuscitation"[Title/Abstract] OR "fluid management"[Title/Abstract] OR "fluid responsiveness"[Title/Abstract] OR "fluid therapy"[Title/Abstract] OR "hemodynamic optimization"[Title/Abstract] OR "clinical outcomes"[Title/Abstract])).

For ScienceDirect, the search strategy was as follows: ("point-of-care ultrasound" OR POCUS) AND ("septic shock") AND (resuscitation OR "fluid resuscitation").

For the Cochrane Library, the following search terms were applied: ("point-of-care ultrasound" OR POCUS OR "ultrasound-guided" OR echocardiography OR "lung ultrasound" OR "integrated cardiopulmonary ultrasound" OR VExUS OR "IVC ultrasound") AND ("septic shock" OR sepsis OR "severe sepsis") AND (resuscitation OR "fluid management" OR "fluid responsiveness" OR "hemodynamic optimization" OR "fluid therapy").

In Google Scholar, a simplified combination of the above keywords was used, and the first 400 results sorted by relevance were screened to identify additional eligible studies. Reference lists of all included articles and relevant reviews were also manually screened to capture any further studies not identified through the electronic database searches.

Eligibility Criteria

Studies were included if they enrolled adult patients (≥18 years) with sepsis or septic shock, defined according to contemporaneous international criteria used in the original studies, most commonly suspected or confirmed infection with organ dysfunction (elevated Sequential Organ Failure Assessment {SOFA} score) and septic shock characterized by persistent hypotension (mean arterial pressure {MAP}: <65 mmHg) requiring vasopressors and/or hyperlactatemia (lactate: ≥2-4 mmol/L) despite initial fluid resuscitation. The intervention consisted of point-of-care ultrasound (POCUS)-guided resuscitation or hemodynamic management, including the assessment of IVC respiratory variability; passive leg raising combined with transthoracic echocardiography (TTE)-derived stroke volume or cardiac output changes; lung ultrasound (LUS) B-line scores; integrated cardiopulmonary ultrasound (ICUS) evaluating cardiac function, preload, and extravascular lung water; or venous excess ultrasound (VExUS) grading of systemic venous congestion. In several studies, ultrasound findings were integrated with advanced hemodynamic monitoring (e.g., pulse contour cardiac output {PiCCO}) to guide fluid therapy, vasopressor titration, and inotropic support. The comparison group received conventional resuscitation, typically based on Surviving Sepsis Campaign recommendations or early goal-directed therapy (EGDT), using predefined targets such as static fluid boluses, central venous pressure (CVP), MAP goals, urine output, and clinical examination, without systematic ultrasound guidance.

The outcomes of interest included mortality (in-hospital, ICU, 28-day, or 30-/90-day), hemodynamic stabilization (achievement of MAP of ≥65 mmHg, vasopressor dose and duration, and time to shock resolution), tissue perfusion markers (lactate levels and lactate clearance and central venous oxygen saturation {ScvO₂}), fluid-related outcomes (total fluid volume, cumulative fluid balance, and pulmonary edema), organ dysfunction (SOFA score changes, acute kidney injury {AKI}, and the need for renal replacement therapy), and resource utilization (duration of mechanical ventilation and ICU and hospital length of stay). Eligible studies included randomized controlled trials, prospective or retrospective cohort studies, and case-control studies. Studies were excluded if they involved pediatric populations (<18 years); were reviews, editorials, case reports, or conference abstracts; or did not include POCUS as a core component of resuscitation or hemodynamic decision-making.

Study Selection

All records retrieved from the databases were compiled into a single dataset, and duplicate entries were removed by using Rayyan AI (Rayyan Systems Inc., Cambridge, MA). Two independent reviewers screened all titles and abstracts to identify potentially relevant studies. Full-text articles of selected studies were then assessed for eligibility based on predefined inclusion and exclusion criteria. Any discrepancies between reviewers were resolved through discussion or consultation with a third reviewer to ensure consistency and objectivity in study selection. The selection process was documented using a PRISMA 2020 flow diagram.

Data Extraction

Data extraction was performed independently by two reviewers using a standardized data extraction template. The extracted information included study identifiers (author, year, and country), study design, sample size, patient characteristics, intervention and comparator details, illness severity scores (SOFA or Acute Physiology and Chronic Health Evaluation II {APACHE II}), and primary and secondary outcomes such as mortality, lactate clearance, mean arterial pressure (MAP), vasopressor duration, total fluid balance, and ICU or hospital length of stay. Extracted data were cross-checked for consistency, and discrepancies were resolved by consensus. The data were then synthesized descriptively to allow structured comparison across studies.

Risk of Bias Assessment

The methodological quality of randomized controlled trials was evaluated using the Cochrane Risk of Bias 2.0 tool, assessing domains such as randomization process, deviations from intended interventions, missing data, outcome measurement, and selective reporting [17]. Observational studies were assessed using the Newcastle-Ottawa scale (NOS), focusing on selection, comparability, and outcome domains [18].

Data Synthesis

A narrative synthesis was conducted due to significant heterogeneity across the included studies in design, patient populations, ultrasound-guided protocols (e.g., IVC, LUS, TTE, and VExUS), comparator groups, and outcome reporting. Variations in the timing and measurement of endpoints such as mortality, hemodynamic parameters, lactate clearance, fluid balance, and organ dysfunction precluded a meta-analysis. Therefore, results were summarized descriptively across five outcome domains: mortality, hemodynamic and perfusion parameters, fluid management, organ dysfunction, and ICU outcomes.

To enhance interpretability, the certainty of evidence was assessed using the Grading of Recommendations, Assessment, Development, and Evaluation (GRADE) approach adapted for narrative syntheses. Certainty was evaluated across risk of bias, inconsistency, indirectness, imprecision, and publication bias and classified as high, moderate, low, or very low for each outcome domain. Findings were then synthesized descriptively across five prespecified domains: mortality, hemodynamic and perfusion parameters, fluid management, organ dysfunction, and ICU-related outcomes.

Results

**Figure 1:**
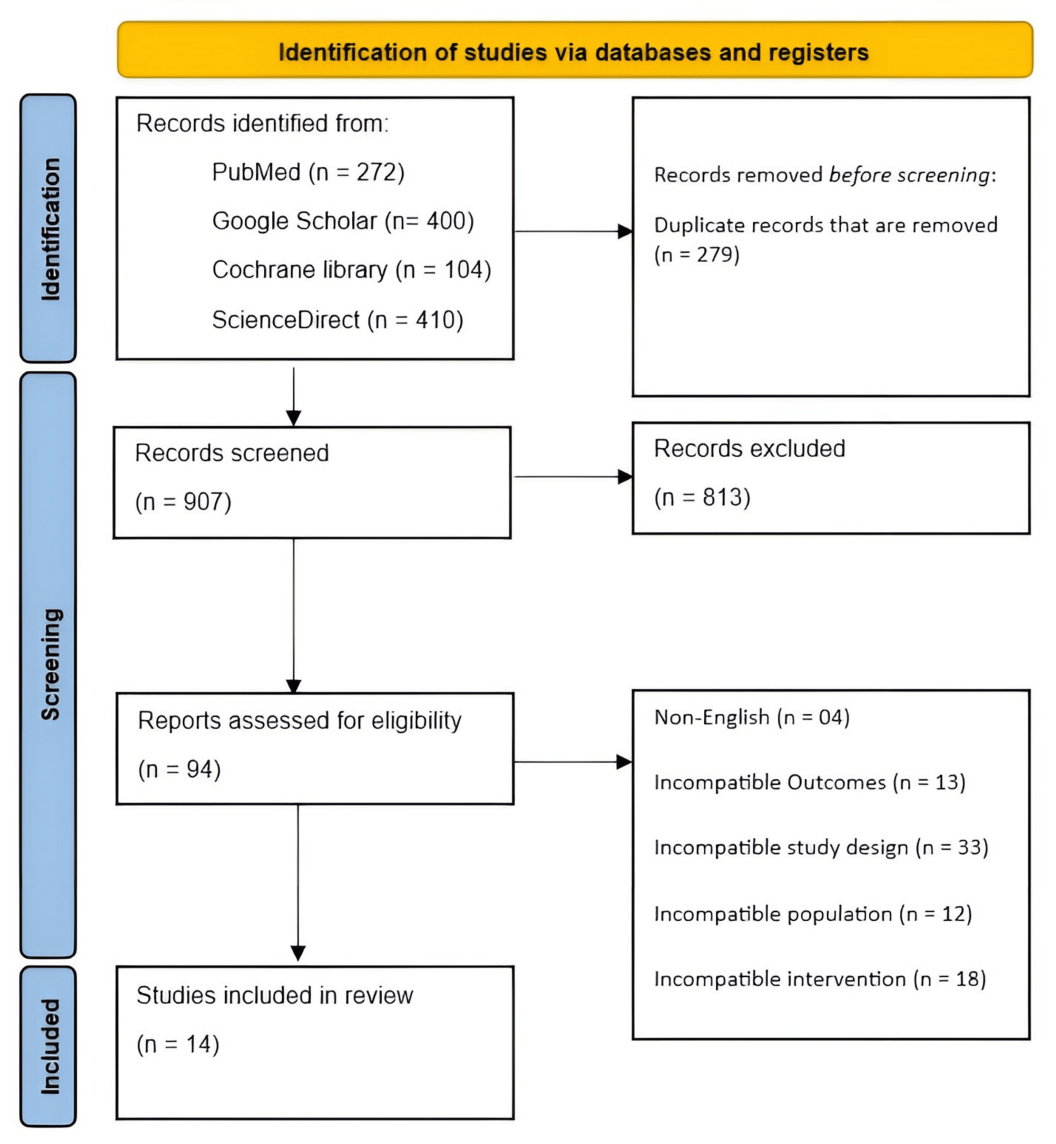
PRISMA flow diagram. PRISMA: Preferred Reporting Items for Systematic Reviews and Meta-Analyses

The literature search identified a total of 1,186 records from electronic databases, including PubMed (n = 272), Google Scholar (first 400 records), the Cochrane Library (n = 104), and ScienceDirect (n = 410). After the removal of 279 duplicate records, 907 records remained and were screened based on titles and abstracts. Of these, 813 records were excluded for not meeting the inclusion criteria. The full texts of 94 articles were subsequently assessed for eligibility. Following full-text review, 80 studies were excluded due to non-English language (n = 4), incompatible outcomes (n = 13), incompatible study design (n = 33), incompatible population (n = 12), or incompatible intervention (n = 18). Ultimately, 14 studies met all eligibility criteria and were included in the systematic review (Figure 1).

Study Characteristics

A total of 14 studies, published between 2018 and 2025, were included in this systematic review, comprising 10 randomized controlled trials (including feasibility, single-center, and single-blinded designs), two prospective cohort studies, one retrospective observational cohort study, and one randomized case-control trial. These studies evaluated the impact of point-of-care ultrasound (POCUS)-guided resuscitation protocols on hemodynamic outcomes and mortality in adult patients with sepsis or septic shock, including three studies conducted primarily in emergency department (ED) settings and 11 studies conducted in intensive care unit (ICU) settings.

Sample sizes ranged from 30 to 304 participants, with most studies enrolling critically ill adult patients diagnosed with sepsis-induced tissue hypoperfusion or septic shock. The reported patient age was predominantly in the sixth to seventh decade of life, with mean or median ages ranging from approximately 54 to 69 years across studies. Where reported, male patients constituted the majority, with male-to-female ratios generally ranging from 55:45 to 65:35.

The interventions primarily included ultrasound-guided fluid resuscitation strategies based on the assessment of the inferior vena cava (IVC) diameter, lung ultrasound B-line score, echocardiography, or integrated cardiopulmonary ultrasound (ICUS), compared with standard care or early goal-directed therapy (EGDT) (Table 1).

**Table 1:** Characteristics of the studies included. APACHE II, Acute Physiology and Chronic Health Evaluation II; SOFA, Sequential Organ Failure Assessment; ED, emergency department; IVC, inferior vena cava; SBP, systolic blood pressure; POCUS, point-of-care ultrasound; USER, Ultrasound Sepsis Resuscitation; BP, blood pressure; PiCCO, pulse contour cardiac output; ScvO₂, central venous oxygen saturation; NE, norepinephrine; RRT, renal replacement therapy; AKI, acute kidney injury; IV, intravenous; CHF, congestive heart failure; HR, heart rate; VExUS, venous excess ultrasound; COPD, chronic obstructive pulmonary disease

Author (Year)	Study Design	Mean/Median Age (Years)	Male/Female (n)	Population Type and Number	Intervention	APACHE II/SOFA Score	Outcomes Assessed	Common Comorbidities Reported
Musikatavorn et al., 2021 [19]	Randomized controlled trial	64.5 ± 18.5 (total cohort)	116/86 (total cohort)	Adult patients with sepsis-induced tissue hypoperfusion (SITH) and septic shock in an ED (n = 202) (101 per group)	Ultrasound-guided fluid management (UGFM) using respiratory change in IVC diameter versus usual care	SOFA: ~6.5 (similar between groups)	30-day mortality, lactate clearance, change in SOFA, hospital length of stay (LOS), and 24-hour fluid volume	Not reported (NR)
Ablordeppey et al., 2024 [20]	Retrospective observational cohort	Median: 65	170/134	Adult ED patients with septic shock (SBP < 90 or mean arterial pressure {MAP} < 65 and lactate ≥ 4) (n = 304) (78 with POCUS and 226 without)	POCUS-guided fluid assessment versus no POCUS	Not reported; severity by lactate ≥ 4; hypotension	Total IV fluid volume (mL/kg), new oxygen requirement, and ED/hospital mortality	CHF and/or severe renal disease (42% POCUS; 29% non-POCUS)
Devia Jaramillo and Menendez Ramirez, 2021 [21]	Prospective cohort study	Median: 65-66	43/40 (approximately)	ED patients with septic shock (n = 83) (USER group versus conventional {CON} group)	USER protocol (ultrasound-guided resuscitation) versus standard care	Protocol U: mean of 5.89 and SD of 2.23; protocol C: mean of 5.95 and SD of 2.27	In-hospital mortality, total and hourly fluid balance, time to BP recovery, and norepinephrine initiation	All patients with septic shock and similar SOFA scores
Yao et al., 2021 [22]	Randomized controlled trial	Median: 65	NR	68 patients with septic shock	Transabdominal ultrasound + PiCCO-guided fluid resuscitation versus PiCCO alone	Not specified	Blood lactic acid (BLA), central venous pressure (CVP), oxygenation index, ScvO₂, extravascular lung water index (EVLWI), mechanical ventilation (MV) time, hospital stay, and survival	Patients in the ICU with septic shock; comorbidities not specified; clinical parameters reported: APACHE II, SOFA, WBC, C-reactive protein (CRP), BLA, and ScvO₂
Li et al., 2021 [23]	Randomized controlled trial	Integrated cardiopulmonary ultrasound (ICUS), 54.5 ± 15.2; CON, 56.7 ± 11.0	ICUS, 24/25; CON, 28/17	94 patients with septic shock (ICUS group, n = 49; CON group, n = 45)	Integrated cardiopulmonary ultrasound (ICUS) for hemodynamic decision-making within one hour versus standard treatment	ICUS group and CON group (APACHE II, 20.8 ± 8.2 versus 21.8 ± 6.7; SOFA, 13.2 ± 4.8 versus 13.2 ± 4.1)	28-day mortality; fluid balance at six, 24, and 72 hours; vasopressor use; lactate clearance; ventilation duration; and ICU stay	Diabetes, ICUS of 8.2% and CON of 11.1%; hypertension, ICUS of 16.3% and CON of 20%; chronic pulmonary disease, ICUS of 8.2% and CON of 2.2%; coronary artery disease, ICUS of 10.2% and CON of 2.2%; renal disease, ICUS of 6.1% and CON of 20%; history of operation, ICUS of 10.2% and CON of 24.4%; hepatology: ICUS of 8.2% and CON of 13.3%
Li et al., 2019 [24]	Prospective randomized controlled trial	Mean: ~60 years	Balanced between groups	74 patients with septic shock (passive leg raising {PLR} + transthoracic echocardiography {TTE} group, n = 37; control, n = 37)	Passive leg raising (PLR) + transthoracic echocardiography (TTE)-guided early fluid resuscitation versus traditional rapid fluid replacement	Not specified	Mean arterial pressure (MAP), lactic acid (Lac), PaO₂/FiO₂, ScvO₂, C-reactive protein (CRP), pulmonary edema incidence, hospital stay, and mortality	Not specified; baseline comorbidities balanced
Li et al., 2025 [25]	Randomized controlled trial (single-center and prospective)	Mean: ~64 years	60%, M; 40%, F	113 adults with septic shock in ICU (study, n = 57; control, n = 56)	Critical care ultrasound-guided individualized fluid management versus conventional guideline-based early goal-directed therapy (EGDT)	SOFA score significantly improved post-treatment (p < 0.05)	ScvO₂, HR, MAP, blood lactate, lactate clearance, SOFA score, pulmonary edema, left heart failure, and ICU length of stay	Hypertension, diabetes, and chronic kidney disease (CKD)
Lanspa et al., 2018 [26]	Randomized controlled feasibility trial	Echo, 69 (median, IQR of 61-77); EGDT, 64 (median, IQR of 49-75)	Echo, 8 M/7F; EGDT, 7 M/8 F	30 adult ICU patients with early septic shock (echo, n = 15; EGDT, n = 15)	Echocardiography-guided resuscitation versus modified early goal-directed therapy (EGDT)	Baseline SOFA comparable; median ΔSOFA: -4 (Echo) versus -6 (EGDT)	ΔSOFA at 48 hours, fluid balance, dobutamine use, lactate clearance, mortality, and ICU-free and ventilator-free days	Common ICU comorbidities likely include chronic cardiovascular, renal, or pulmonary conditions, but specifics were not detailed
Fattah et al., 2025 [27]	Randomized case-control trial	Mean: 66.58 ± 12.39	73/39	112 adult sepsis/septic shock patients (56, VExUS; 56, conventional)	VExUS-guided resuscitation versus Surviving Sepsis Campaign (SSC)-guided	Not reported	Mortality; cumulative fluid balance; AKI; need for RRT; respiratory failure (RF); mechanical ventilation (MV); ICU and hospital length of stay	Hypertension (67.9%), diabetes (54.5%), COPD (33%), and chronic kidney disease (27.7%)
Chen et al., 2018 [28]	Prospective observational cohort study	Control, 67.4 ± 9.8; intervention, 64.9 ± 11.8	Control, 56/32; intervention, 23/18	129 adult patients with sepsis (88 control; 41 POCUS group) admitted to the emergency ICU	Routine incorporation of POCUS during morning rounds versus standard care (occasional POCUS)	Not reported	Mortality; duration of mechanical ventilation (MV); ICU length of stay; fluid balance; vasopressor duration	APACHE II score (control, 24.0 ± 4.7; intervention, 26.8 ± 5.1); specific comorbidities not detailed
Zhuang et al., 2020 [29]	Randomized controlled trial	Mean: ~59 years	Not specified; comparable between groups	40 adult patients with septic shock (20, EGDT; 20, ultrasound-guided)	Ultrasound-guided fluid resuscitation using IVC diameter and lung B-line score versus early goal-directed therapy (EGDT)	(SO) 12.2 ± 5.1; 14.3 ± 5	6-hour MAP ≥ 65 mmHg achievement rate; 24-hour total fluid volume; 24-hour NE use; PaO₂/FiO₂; lactic acid clearance rate (LCR); and ICU survival	Underlying diseases (not quantified)
Ismail et al., 2019 [30]	Single-blinded randomized controlled trial	Adult patients, mean/median not specified	Not reported	80 adult patients with septic shock (40, EGDT; 40, ultrasound-guided)	Simplified lung ultrasound (LUS) protocol to determine the endpoint of resuscitation versus EGDT (CVP target: 8-12 mmHg)	Not reported	LUS correlation with hemodynamic and congestion indices; cutoff value for stopping resuscitation	Pneumonia (47.5%. EGDT; 52.5%, ultrasound); other comorbidities not specified
Elsayed Afandy et al., 2020 [31]	Prospective randomized comparative study	Cardiometry, 38.5 ± 3.3; echo, 38.6 ± 3.3; EGDT, 39.4 ± 3.7	Cardiometry, 17/13; echo, 17/13; EGDT, 16/14	90 adult patients with sepsis (30, EGDT; 30, echo-guided; 30, cardiometry-guided)	Transthoracic echocardiography-guided versus electrical cardiometry-guided versus EGDT fluid and vasopressor management	(A) 25.4 ± 3.7; 25.5 ± 3.7	30-day mortality; total infused fluid; vasopressor/inotrope use; MV duration; ICU and hospital stay	Causes of sepsis included abscess, bronchiectasis, endocarditis, peritonitis, and pneumonia. Patients with major cardiac dysrhythmia, severe anemia, renal/hepatic dysfunction, and significant valvular or congenital heart disease were excluded
Alhabashy et al., 2021 [32]	Randomized controlled trial (single-center)	18-60 (matched groups; mean not specified)	59%, M/41%, F	100 adult patients with severe sepsis or septic shock (analyzed: 87; EGDT = 45 and echo = 42)	EGDT group versus echo-guided resuscitation protocol using echocardiographic parameters for hemodynamic management	Not reported	30- and 90-day mortality; total fluid intake (first 24 hours); vasopressor and inotrope use; time to normalization of hemodynamics; time to vasopressor weaning	Excluded known cardiac disease; other comorbidities not reported

Commonly reported comorbidities included hypertension, diabetes mellitus, chronic kidney disease, chronic pulmonary disease, coronary artery disease, and congestive heart failure. The severity of illness was reported using SOFA or APACHE II scores in several trials, which generally indicated moderate to severe septic shock (SOFA, 6-13; APACHE II, 20-25). Across studies, the primary outcomes included mortality (in-hospital, 28-day, or 30-day), lactate clearance, hemodynamic stabilization (MAP ≥ 65 mmHg), vasopressor duration, fluid balance, and ICU or hospital length of stay (Table 1).

Quality Assessment

**Figure 2:**
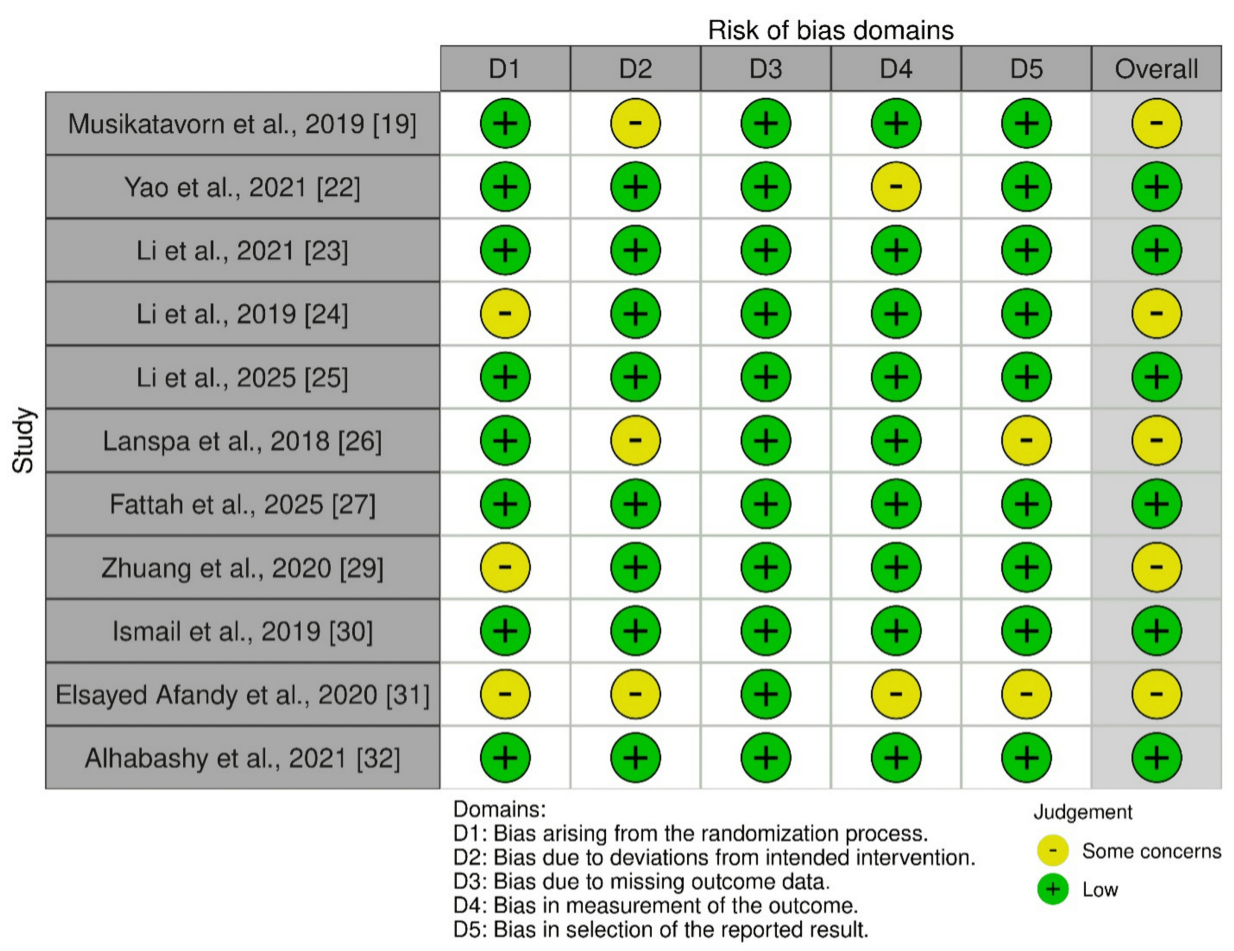
Quality assessment of RCTs by RoB 2.0. Risk of bias assessment across D1-D5 domains. Adapted from the Cochrane Risk of Bias (RoB) tool version 2.0 [17]. RCTs: randomized controlled trials

Quality assessment indicated that most randomized controlled trials exhibited an overall low to moderate risk of bias. Domain-specific evaluation revealed that the randomization process (D1) was generally well described; however, some studies lacked sufficient detail regarding allocation concealment, resulting in some concerns. Deviations from intended interventions (D2) represented a frequent limitation, primarily due to the absence of blinding of clinicians and participants, which is inherently challenging in ultrasound-guided resuscitation protocols. Missing outcome data (D3) and outcome measurement (D4) were largely robust across studies, with minimal attrition and objective outcome measures, contributing to a low risk of bias in these domains. In contrast, the selection of reported results (D5) raised concerns in a subset of trials owing to the incomplete reporting of prespecified outcomes or the lack of protocol registration (Figure 2).

Among the observational cohort studies, quality assessment using the Newcastle-Ottawa scale demonstrated that two studies were of moderate quality and one of high quality. Deficiencies were mainly related to the comparability of cohorts, with limited adjustment for confounding variables, and outcome assessment, where follow-up duration or outcome ascertainment methods were insufficiently detailed. Overall, despite these limitations, the included studies demonstrated acceptable methodological rigor, supporting the reliability of the synthesized evidence (Table 2).

**Table 2:** Quality assessment of cohort studies by the Newcastle-Ottawa scale. Source: [18].

Author (Year)	Selection (Max Four)	Comparability (Max Two)	Outcome (Max Three)	Total Score (Max Nine)	Quality Rating
Ablordeppey et al., 2024 [20]	3	1	2	6	Moderate
Devia Jaramillo and Menendez Ramirez, 2021 [21]	3	2	3	8	High
Chen et al., 2018 [28]	3	1	2	6	Moderate

Mortality Outcomes

Mortality outcomes varied across studies, with heterogeneous effect sizes and limited consistency in clinically meaningful benefit. Musikatavorn et al. reported nearly identical 30-day mortality between ultrasound-guided and standard care groups (19.8% versus 18.8%; absolute risk difference {ARD}: 1.0%), indicating no clinically relevant survival advantage [19]. Similarly, Ablordeppey et al. observed hospital mortality rates of 31% versus 27% (ARD: 4%), a difference that was neither statistically nor clinically significant given baseline severity imbalance favoring the control group [20]. Devia Jaramillo and Menendez Ramirez found in-hospital mortality of 56.4% versus 61.3% (ARD: -4.9%), suggesting a modest but non-significant trend toward benefit [21].

In contrast, Alhabashy et al. demonstrated a clinically meaningful reduction in mortality, with 30-day mortality decreasing from 35.6% to 14.3% (ARD: -21.3%) and 90-day mortality from 40% to 16.7% (ARD: -23.3%), indicating a substantial survival advantage with echocardiography-guided resuscitation [32]. Elsayed Afandy et al. similarly reported improved survival with echo- and cardiometry-guided strategies compared to EGDT, although exact effect estimates were not fully reported, limiting the interpretation of clinical magnitude [31]. Other trials, including those by Li et al. [23], Li et al. [24], Zhuang et al. [29], and Lanspa et al. [26], showed minimal or no absolute mortality differences, suggesting that while physiological improvements were evident, these did not consistently translate into survival benefit. Fattah et al. reported a mortality reduction from 51.8% to 42.9% (ARD: -8.9%), representing a potentially clinically relevant effect that did not reach statistical significance, possibly due to limited sample size [27]. Overall, mortality benefits appear clinically meaningful in select trials but remain inconsistent across the evidence base.

Hemodynamic Parameters and Lactate Clearance

Across studies, ultrasound-guided resuscitation was associated with clinically relevant improvements in perfusion markers, particularly lactate reduction and oxygenation. Yao et al. demonstrated significantly lower blood lactate levels and higher ScvO₂ in the ultrasound + PiCCO group, accompanied by shorter ventilation duration and hospital stay, supporting both physiological and clinical relevance [22]. Li et al. reported improved MAP and ScvO₂ alongside higher six-hour lactate clearance, reflecting faster shock reversal rather than isolated statistical effects [25]. Li et al. observed a mean lactate reduction of approximately 1.4 mmol/L and improved PaO₂/FiO₂ by ~35 mmHg, changes that are clinically meaningful in septic shock management [24]. In contrast, Musikatavorn et al. found no appreciable difference in SOFA change or lactate clearance, suggesting that isolated IVC-based assessment may offer limited clinical impact when not integrated into a comprehensive ultrasound protocol [19].

Fluid Management and Balance

Most studies demonstrated clinically important reductions in fluid exposure, which are directly relevant to preventing fluid overload complications. Musikatavorn et al. [19] and Devia Jaramillo and Menendez Ramirez [21] reported reductions of approximately 400-700 mL within the first 6-24 hours, a magnitude likely to influence pulmonary and renal outcomes. Zhuang et al. [29] observed a reduction of nearly 900 mL in 24-hour fluid volume, while Chen et al. [28] reported a shift from positive to negative daily fluid balance (-143 versus +48 mL), changes associated with reduced ICU stay. Fattah et al. demonstrated a net difference of ~3 L in cumulative fluid balance, accompanied by marked reductions in AKI and respiratory failure, highlighting clear clinical relevance beyond statistical significance [27].

Organ Dysfunction and SOFA Score

Improvements in organ dysfunction were clinically meaningful in several studies. Li et al. reported significant SOFA score reductions, alongside reduced pulmonary edema and left heart failure, indicating global organ recovery rather than isolated score changes [25]. Devia Jaramillo and Menendez Ramirez showed a 47% absolute increase in achieving MAP of ≥65 mmHg within six hours, reflecting faster hemodynamic stabilization [21]. Li et al. reported lower C-reactive protein (CRP) levels and a 24% absolute reduction in pulmonary edema incidence, reinforcing the clinical impact of ultrasound-guided strategies [24].

ICU, Mechanical Ventilation, and Hospital Outcomes

Reductions in ICU stay and ventilation duration were both statistically and clinically relevant in several trials. Fattah et al. reported a reduction in mechanical ventilation duration by nearly three days and ICU stay by over three days, effects with direct implications for resource utilization and patient recovery [27]. Chen et al. [28] demonstrated a 1.2-day reduction in ventilation duration and a 61% lower risk of prolonged ICU stay, while Alhabashy et al. [32] reported shorter ICU and hospital lengths of stay alongside improved survival. Conversely, Musikatavorn et al. [19] and Lanspa et al. [26] showed minimal differences, indicating that not all ultrasound strategies confer comparable clinical benefit (Table 3).

**Table 3:** Comparative summary of clinical outcomes reported in studies evaluating ultrasound-guided versus conventional resuscitation strategies in adult patients with sepsis and septic shock. Ultrasound-guided strategies included inferior vena cava (IVC) assessment, lung ultrasound (LUS), echocardiography, integrated cardiopulmonary ultrasound, passive leg raising with echocardiography, VExUS scoring, and combined ultrasound-hemodynamic monitoring approaches. Comparator strategies consisted of usual care, early goal-directed therapy (EGDT), or Surviving Sepsis Campaign-guided resuscitation. AKI: acute kidney injury; CVP, central venous pressure; ICU, intensive care unit; LOS, length of stay; MAP, mean arterial pressure; MV, mechanical ventilation; RRT, renal replacement therapy; ScvO₂, central venous oxygen saturation; SOFA, Sequential Organ Failure Assessment; VExUS, venous excess ultrasound

Author (Year)	Outcome Domain	Ultrasound-Guided Group	Comparator Group	Key Result
Musikatavorn et al., 2021 [19]	Mortality	30-day mortality: 19.8%	18.8%	No significant difference
	Fluid management	Lower fluid volume at 24 hours	Higher fluid volume	Reduced fluid administration with ultrasound guidance
	Organ dysfunction	No change in SOFA score	No change in SOFA score	Neutral effect
Ablordeppey et al., 2024 [20]	Mortality	Hospital mortality: 31%	27%	No significant difference
	Fluid management	33.0 mL/kg	32.1 mL/kg	No significant difference
Devia Jaramillo and Menendez Ramirez, 2021 [21]	Mortality	56.4%	61.4%	No significant difference
	Hemodynamics	MAP ≥ 65 mmHg achieved in 97.4%	50%	Significant improvement
	Fluid balance	Lower balance at 4-6 hours	Higher balance	Reduced early fluid administration
Yao et al., 2021 [22]	Perfusion	Lower lactate and higher ScvO₂	Less improvement	Significant benefit
	ICU outcomes	Shorter mechanical ventilation and hospital stay	Longer duration	Improved outcomes
Li et al., 2021 [23]	Mortality	No difference at 28 days	-	Neutral
	Hemodynamics	Shorter vasopressor duration	Longer duration	Improved hemodynamic stability
Li et al., 2019 [24]	Perfusion	Lower lactate and higher PaO₂/FiO₂ and ScvO₂	Inferior values	Significant improvement
	Pulmonary edema	13.5%	37.8%	Reduced incidence
Li et al., 2025 [25]	Organ dysfunction	Lower SOFA score and pulmonary edema	Higher values	Significant benefit
	ICU stay	Shorter ICU length of stay	Longer ICU stay	Reduced length of stay
Lanspa et al., 2018 [26]	Mortality	33%	20%	No significant difference
	Organ dysfunction	Similar change in the SOFA score	-	Neutral
Fattah et al., 2025 [27]	Mortality	42.9%	51.8%	Non-significant trend toward benefit
	Fluid balance	-1.58 L	+1.43 L	Significant reduction in cumulative balance
	AKI/RRT	Lower incidence	Higher incidence	Reduced renal complications
Chen et al., 2018 [28]	ICU outcomes	Shorter mechanical ventilation and ICU stay	Longer duration	Improved ICU outcomes
Zhuang et al., 2020 [29]	Fluid management	Lower 24-hour fluid volume	Higher volume	Reduced fluid administration
	Oxygenation	Less deterioration	Greater deterioration	Protective effect
Ismail et al., 2019 [30]	Fluid overload	Lung ultrasound-guided endpoint	CVP-guided endpoint	Prevention of fluid congestion
Elsayed Afandy et al., 2020 [31]	Mortality	Lower mortality	Higher mortality	Improved survival
Alhabashy et al., 2021 [32]	Mortality	Reduced 30- and 90-day mortality	Higher mortality	Significant reduction
	ICU outcomes	Faster hemodynamic stabilization and shorter ICU stay	Longer duration	Improved outcomes

Certainty of Evidence (GRADE Assessment)

Using the GRADE approach adapted for narrative syntheses, the certainty of evidence for ultrasound-guided resuscitation in sepsis was rated as moderate for improvements in hemodynamic stabilization, tissue perfusion (lactate clearance), and fluid management outcomes, reflecting generally consistent effects across randomized and observational studies despite protocol heterogeneity. Evidence for reductions in pulmonary congestion and selected organ dysfunction outcomes (particularly acute kidney injury) ranged from low to moderate, limited by imprecision and single-center study designs. In contrast, the certainty of evidence for mortality reduction was low, due to inconsistent findings and underpowered trials, indicating no clear survival benefit across studies (Table 4).

**Table 4:** GRADE summary of findings for ultrasound-guided resuscitation in sepsis and septic shock. ⊕⊕◯◯: limited confidence; further research is likely to change the estimate. ⊕⊕⊕◯: moderately confident; further research may change the estimate. GRADE, Grading of Recommendations, Assessment, Development, and Evaluation; ED, emergency department; ICU, intensive care unit; RCTs, randomized controlled trials; MAP, mean arterial pressure; ScvO₂, central venous oxygen saturation; LUS, lung ultrasound; VExUS, venous excess ultrasound; SOFA, Sequential Organ Failure Assessment; AKI, acute kidney injury; RRT, renal replacement therapy; MV, mechanical ventilation; LOS, length of stay

Outcome Domain	Number of Studies (Design)	Direction and Consistency of Effect	Key Limitations (GRADE Domains)	Certainty of Evidence (GRADE)	Summary Interpretation
Mortality (ED, ICU; 28-90 days)	11 studies (seven RCTs and four observational)	No consistent mortality benefit across studies; few single-center RCTs reported benefit	Serious inconsistency; imprecision; some risk of bias; underpowered trials	Low, ⊕⊕◯◯	Current evidence does not demonstrate a consistent mortality reduction with ultrasound-guided resuscitation
Hemodynamic stabilization (MAP, ScvO₂, CO, and vasopressor duration)	10 studies (six RCTs and four observational)	Generally favors ultrasound guidance with faster stabilization and reduced vasopressor exposure	Some inconsistency; indirectness due to protocol variability	Moderate, ⊕⊕⊕◯	Ultrasound-guided strategies improve early hemodynamic optimization
Perfusion markers (lactate and lactate clearance)	9 studies (six RCTs and three observational)	Consistent improvement in lactate reduction and clearance in ultrasound-guided groups	Risk of bias in some trials; variable timing of assessment	Moderate, ⊕⊕⊕◯	Ultrasound guidance is associated with improved tissue perfusion markers
Fluid balance and volume administered	12 studies (seven RCTs and five observational)	Consistently reduced fluid volumes and more negative fluid balance	Some indirectness; heterogeneity in protocols	Moderate, ⊕⊕⊕◯	Strong and consistent evidence that ultrasound guidance reduces fluid overload
Pulmonary congestion/edema	7 studies (RCTs and cohorts using LUS and VExUS)	Consistent reduction in pulmonary edema and respiratory complications	Small sample sizes; single-center studies	Moderate, ⊕⊕⊕◯	Ultrasound-guided resuscitation reduces fluid-related pulmonary complications
Organ dysfunction (SOFA, AKI, and RRT)	6 studies (four RCTs and two observational)	Directionally favors ultrasound guidance; strongest for AKI reduction	Imprecision; some risk of bias	Low-moderate, ⊕⊕◯◯ to ⊕⊕⊕◯	Ultrasound guidance may reduce organ dysfunction, particularly renal injury
ICU outcomes (MV duration, ICU LOS, and hospital LOS)	8 studies (five RCTs and three observational)	Generally shorter MV duration and ICU stay	Inconsistency; indirectness	Low-moderate, ⊕⊕◯◯ to ⊕⊕⊕◯	Likely benefit in ICU resource utilization, though certainty is limited

Discussion

This systematic review evaluated the impact of ultrasound-guided fluid management on clinical outcomes in patients with sepsis and septic shock. Across studies, ultrasound-guided strategies, whether using inferior vena cava (IVC) dynamics, transthoracic echocardiography (TTE), venous excess ultrasound (VExUS), or integrated cardiopulmonary ultrasound (ICUS), consistently led to more judicious fluid use compared to standard guideline-based care. Multiple randomized controlled trials (RCTs) reported significantly lower cumulative fluid balance in ultrasound groups, suggesting that dynamic fluid responsiveness assessment helps prevent unnecessary volume expansion and related complications such as pulmonary edema and respiratory failure [21,24,27-29,32]. This trend was observed in trials using IVC measurements and lung ultrasound [29,30], as well as in studies employing more comprehensive hemodynamic tools such as VExUS and ICUS [23,27]. The reduction in fluid volume was often accompanied by faster hemodynamic stabilization, reflected by the earlier achievement of target mean arterial pressure (MAP) and earlier vasopressor weaning [21,32].

Mechanistically, these benefits likely stem from ultrasound's ability to differentiate true preload responsiveness from vasoplegic shock or septic cardiomyopathy, clinical states in which additional fluid administration produces minimal stroke volume augmentation while increasing venous congestion and interstitial edema [24,26,29,30]. Trials employing dynamic assessments, such as passive leg raising combined with TTE or respiratory IVC variability, more effectively identified patients positioned on the ascending limb of the Frank-Starling curve, thereby converting administered fluid into meaningful hemodynamic gains [24,29]. In contrast, protocol-driven or static resuscitation strategies risked indiscriminate volume expansion, particularly in patients with preserved preload but impaired myocardial function [26,31].

These results align with the findings of Chen et al., whose meta-analysis of 12 RCTs (n = 947) concluded that ultrasound-guided fluid resuscitation in septic shock significantly reduced mortality (RR, 0.78; 95% CI, 0.65-0.94), total 24-hour fluid volume, and ICU/hospital length of stay while improving hemodynamic efficiency through tools such as passive leg raising combined with echocardiography [33].

Several studies demonstrated improvements in tissue perfusion and organ function parameters, including higher ScvO₂, better oxygenation indices, and enhanced lactate clearance in ultrasound-guided groups [22-25,29]. These physiological benefits were most evident in trials integrating ultrasound with advanced hemodynamic monitoring (e.g., PiCCO), where the real-time visualization of cardiac output, extravascular lung water, and venous return enabled tighter coupling between resuscitation interventions and tissue-level perfusion responses [22,25]. By limiting excessive fluid loading and pulmonary capillary leak, ultrasound-guided strategies plausibly improved ventilation-perfusion matching and oxygen delivery, thereby accelerating lactate clearance without increasing respiratory complications [22,24,29].

Additionally, ultrasound-guided approaches often resulted in lower rates of acute kidney injury (AKI) and shorter mechanical ventilation duration, likely due to the avoidance of excessive fluid loading [27,28]. VExUS-guided trials provided particularly strong mechanistic insight, demonstrating that higher venous congestion grades correlated with worse renal outcomes, respiratory failure, and mortality [27]. These findings support the concept that venous congestion, rather than absolute hypovolemia alone, is a critical driver of organ dysfunction in septic shock and that ultrasound-guided decongestion strategies may mitigate cardiorenal and pulmonary injury [27,30].

Similarly, Basmaji et al., in a large systematic review and meta-analysis of 18 RCTs, found that point-of-care ultrasound (POCUS)-guided resuscitation probably reduces 28-day mortality (RR, 0.88; 95% CI, 0.78-0.99), shortens vasopressor duration (mean difference: -0.73 days), and lowers the need for renal replacement therapy (RR, 0.80; 95% CI, 0.63-1.02), thereby reinforcing the evidence that ultrasound-guided management yields clinically meaningful improvements in shock resuscitation outcomes [15].

Despite these physiological and resource-related benefits, most studies did not demonstrate a statistically significant reduction in mortality. Mortality outcomes were similar between ultrasound-guided and conventional resuscitation groups in several RCTs and cohort studies [19,21,24,26].

This apparent discordance between improved physiological endpoints and neutral mortality outcomes likely reflects several mechanistic and contextual factors: (1) early aggressive pre-enrollment resuscitation reducing group separation, as observed by Lanspa et al. [26]; (2) relatively lower baseline illness severity in some cohorts (e.g., SOFA ≈ 6 by Musikatavorn et al. [19]), limiting the capacity to detect mortality differences; and (3) ultrasound protocols that primarily restricted fluids without systematically optimizing vasopressor or inotropic support, potentially attenuating survival benefit [21,23,31]. A few trials, however, such as by Alhabashy et al., observed significant mortality reductions at both 30 and 90 days with echo-guided resuscitation, and others showed non-significant trends toward improved survival [32].

Notably, mortality-positive trials were characterized by early ultrasound deployment, repeated reassessment, and the protocolized integration of echocardiographic findings into vasopressor and inotrope titration, suggesting that ultrasound confers maximal benefit when embedded within a structured decision-making framework rather than applied as a one-time diagnostic tool [27,32]. The meta-analyses by Basmaji et al. [15] and Chen et al. [33] reconcile these inconsistencies by showing pooled evidence of mortality reduction with ultrasound guidance, though both emphasize that the magnitude of benefit is likely contingent upon operator expertise, the timing of intervention, and protocol standardization.

Importantly, the integration of ultrasound into early sepsis management protocols appears feasible and clinically valuable. Studies conducted in emergency departments and ICUs alike have shown that ultrasound-guided approaches can be effectively implemented within the early resuscitation phase [19,28]. The benefits were especially apparent when ultrasound was used repeatedly during resuscitation or combined with clinical and laboratory indices rather than as a single-point assessment. Moreover, correlations between ultrasound-derived congestion scores (e.g., lung ultrasound score and VExUS grade) and organ dysfunction severity (e.g., SOFA score) suggest that ultrasound provides a dynamic and noninvasive surrogate marker of evolving pathophysiology [27,30]. This supports the mechanistic premise that septic shock represents a heterogeneous and time-sensitive syndrome, in which the continuous reassessment of preload responsiveness, cardiac function, and venous congestion is superior to fixed resuscitation targets [23,27,30].

This systematic review has several limitations. First, significant heterogeneity existed among the included studies in terms of patient populations, ultrasound modalities, timing, and operator expertise. Importantly, this heterogeneity likely reflects true biological variability in septic shock phenotypes, ranging from hypovolemic-dominant states to myocardial depression and venous congestion, each of which may respond differently to ultrasound-guided interventions. Other limitations include small sample sizes, variable methodological quality, inconsistent resuscitation endpoints, and a lack of standardized POCUS competency reporting. Publication bias could not be excluded. Future research should prioritize large, multicenter RCTs using standardized ultrasound-guided resuscitation algorithms, predefined operator competency criteria, and phenotype-stratified analyses to better delineate which patients derive the greatest survival and organ-protective benefit from ultrasound-guided shock management.

## Conclusions

This systematic review demonstrates that point-of-care ultrasound-guided resuscitation protocols enhance the management of septic shock by enabling more accurate hemodynamic assessment and individualized fluid therapy. Ultrasound integration improves perfusion parameters, optimizes fluid balance, and reduces organ dysfunction and ICU stay duration. Although mortality benefits remain inconsistent across studies, trends indicate better clinical outcomes with tailored ultrasound-guided approaches compared to standard care. These findings support incorporating bedside ultrasound as a core component of sepsis resuscitation strategies to achieve more precise, physiologically guided treatment and potentially improve overall patient recovery in critical care settings.
